# Corrigendum: Asymmetric parental genome engineering by Cas9 during mouse meiotic exit

**DOI:** 10.1038/srep44342

**Published:** 2017-06-01

**Authors:** Toru Suzuki, Maki Asami, Anthony C. F. Perry

Scientific Reports 4: Article number: 7621; 10.1038/srep07621 published online: 12 23 2014; updated: 06 01 2017.

This article was originally published under a CC BY-NC-SA 4.0 license, but has now been made available under a CC BY 4.0 license. The PDF and HTML versions of the paper have been modified accordingly.

